# Comparison of CBF1, CBF2, CBF3 and CBF4 expression in some grapevine cultivars and species under cold stress

**DOI:** 10.1016/j.scienta.2015.10.011

**Published:** 2015-12-14

**Authors:** Maryam Karimi, Ali Ebadi, Seyed Amir Mousavi, Seyed Alireza Salami, Abdolkarim Zarei

**Affiliations:** a University of Tehran, Iran; b National Institue of Genetic Engineering and Biotechnology, Iran; c Jahrom University, Iran

**Keywords:** Grape, Transcription factors, Cold stress, Gene expression, CBF genes

## Abstract

Grapevine, an important horticultural crop in the world, is moderately tolerant to cold conditions and is subjected to the cold injuries at different regions. So studies on different aspects of tolerance mechanism to unexpected cold of late spring as well as winter freezing seems necessary about this vine. For this reason, study on genes responsible for acquiring cold tolerance is very important. Transcription factors are among regulatory proteins that are responsible for cold acclimation. In this research work, expression levels of CBF1, CBF2, CBF3, and CBF4 transcription factors were studied on two cvs of *Vitis vinifera* (“Khalili-Danedar” and “Shahroodi”) as well as one *Vitis riparia* at different times after treating at 4 °C. Results showed that two *vinifera* cultivars, “Khalili-Danedar” and “Shahroodi”, had similar trend for each transcription factor. Gene expression increased at the beginning of cold stress and then decreased. Expression of these TF started some minutes (CBF1) after cold treatment and continued for several hours (CBF2), even till the tenth day (CBF4). All together *V. riparia* which is endemic to the cold regions behaved stronger and showed higher expression for all studied transcription factors. Among two *V. vinifera* cultivars, “Khalili-Danedar” showed significantly higher expression compared with “Shahroodi”. The comparison of expression levels of these four transcription factors revealed that the least and the greatest expressions were recorded for CBF1 and CBF3 respectively, and two CBF2 and CBF4 had approximately the same expression levels.

## Highlights

• We compare three genotype after cold stress.
• We found that CBFs have vital rule in cold resistance.
• CBFs have different trend expression for each genotype.
• Riparia and Khalili-Danehdar had high CBFs expression compare with Shahroodi.

## 1 Introduction

Low temperature is one of the important environmental limiting factors that affect distribution and production of agricultural products. Unexpected early autumn freezing and cold of late spring as well as winter freezing will cause significant damages to the plants. In responses to the stressful conditions, plant show a set of morphological, physiological, biochemical and molecular changes, making them more tolerant to survive in these conditions. When a plant faces an abiotic stress, a set of genes with different function will be induced or suppressed. Protein products of these genes act functionally or regulatory in response to these stresses. Evaluating the roles of these genes is required to understand the mechanisms that plants apply to minimize the effects of abiotic stresses as well as for genetic manipulation of plant to increase plant tolerance to stresses (Agarwal et al., 2006, Lata and Prasad, 2011, Shinozaki and Yamaguchi-Shinozaki, 2007). The manipulation of a protein coding gene may be beneficial to induce some tolerance but it is not necessary to exert sufficient tolerance for most abiotic stresses. Therefore, another group of genes, regulatory protein coding genes, is among the targets of genetic engineering. Expression levels of many abiotic stress responsive genes are affected by manipulating the regulatory genes. Therefore, by manipulating these genes it would be possible to manage plant behavior in stress conditions (Thomashow, 2001, Century et al., 2008, Yang et al., 2011). CBF proteins (C1 repeat binding elements), a member of regulatory protein group, have a vital role in plants responses to abiotic stresses (Sakuma et al., 2002).

DREB1/CBF transcription factors are present in many cold resistance plants such as Rapeseed (Jaglo et al., 2001) and Barely (Choi et al., 2002) as well as cold sensitive ones, Tomato and Rice (Dubouzet et al., 2003, Zhang et al., 2004). Some of the trees that show high levels of cold resistance capacity have been screened for DREB1/CBF (Puhakainen et al., 2004) and their orthologous genes are identified in some of these plants such as Birch (Welling and Palva, 2008), Eucalyptus (Navarro et al., 2009) and Grape (Xiao et al., 2006).

It is reported that cold treated *Arabidopsis* plants showed higher expression of CBF1, CBF2 and CBF3 genes, while salinity and drought treatments had no such effects (Gilmour et al., 1998, Liu et al., 1998).

Studies in different plants revealed that CBF transcription factors show different responses when exposed to the environmental stresses (salinity, drought, and cold). Some of DREB1/CBF genes like BrCBF in non-heading Chinese Cabbage (Jiang et al., 2011), MbDREB1 in Apple (Yang et al., 2011), OsDREB1F in Rice (Wang et al., 2008), and VviDREB1 in Cowberry (Wang et al., 2010) respond not only to cold treatment but also to high salinity, drought and exogenous ABA. Interaction between common elements is needed for perceiving abiotic stresses in a cell. It is likely that CBF genes act as the joining points for several pathways and connect salinity, drought, ABA and cold pathways (Qin et al., 2007). In Grapevine, CBF4 gene is often induced by cold treatment, while CBF1, CBF2 and CBF3 respond better to drought (Xiao et al., 2008).

DREB1/CBF proteins have two DNA binding domains (AP2) that specifically attach to the CRT/DRE sequences and activate transcription of the genes downstream of these sequences. In addition to CRT/DRE elements, ICE1 (inducer CBF expression), HOS1 (High expression of somatically responsive gene1) and MYB15 regulate the expression of downstream DREB1/CBF genes. ICE1 is transcription activators of CBF3, which code a constitutive nuclear protein that attaches to MYC elements (CANNTG) in the promoter of CBF3 genes, hence increasing its expression (Chinnusamy et al., 2004). Also, CBF2 mutants (which disturb this gene expression) analysis, indicated down-regulatory effect of this gene on CBF1 and CBF3. Expression level of CBF/DREB1 genes is somehow controlled by themselves (Novillo et al., 2004). Altogether these three proteins act as a network and interact to regulate expression levels of CBF genes and plant responses to cold conditions.

Cultivars in the *Vitis vinifera* species, tolerate gradual cold and are not damaged by winter temperature up to −15 °C, while Asian and northern American species can persist up to −35 °C/−40 °C (Mullins et al., 1992, Fennell, 2004). In the winter of 2007, Iran encountered a cold condition that caused cold damage in a lot of fields and orchard. Most of the accessions in the vineyard collection of University of Tehran were severely damaged or even died, but only a few grapevine cultivars survived and grew next spring. In the present study, expression level of four transcription factors CBF1, CBF2, CBF3, and CBF4 genes, involved in the activation of cold resistance genes was evaluated using quantitative method in the two *V. vinifera* (“Khalili-Danedar” and “Shahroodi”) as well as *Vitis riparia* to understand more about differences in the cold tolerance of these two species.

## 2 Materials and methods

Grapevine cuttings from “Khalili-Danedar” (a cultivar that showed tolerance to the cold and grew the spring after cold stress) and “Shahroodi” (a cultivar that did not show cold tolerance and had regrowth from the root after cold stress) as well as *V. riparia* cuttings were collected from the Grapevine Collection of Horticulture Research at University of Tehran and were rooted. One year old plants were transferred to a phytotron under 4 °C and 20–25 nM/M^2^ S light intensity. At defined times (0.5, 4, 8, 24, 72, 120 and 240 h), leaves were sampled, dipped in liquid nitrogen and stored at −80 °C. RNA was extracted using the method previously described by Reid et al. (2006) for extraction from grape berry with some modifications.

Primers were designed based on the recorded information in the NCBI and Genoscop that have complete information about grape genome sequence.

These four genes have very similar sequences, therefore, to prevent redundancy, we needed to select small regions of sequences with lower similarity during primer designing process. Vector NTI software was used to design primers (Table 1). Primers annealing temperatures were checked using OligoCalc software.

Real time PCR (Applied Biosystems) was used for quantitative PCR analysis. The amplification reactions contained 12 μl SYBR Green PCR Master Mix (Applied Biosystems), 50 ng cDNA, 1.34 μM of each forward and reverse primer in a final reaction volume of 24 μl and performed as follow: 50 °C for 2 min; 95 °C for 10 min; and 40 cycles of 95 °C for 15 s and 60 °C for 1 min and were performed at least in triplicate for each sample. Specific Elongation factor 1 α (EF1α) was used as reference gene. Relative quantitative expression was determined using 2 ^(−ΔΔCt)^ method (Livak and Schmittgen, 2001). The analysis of variance (ANOVA) was performed using the software SAS (ver. 9.1) and graph was constructed using Excel (2013) software.

## 3 Results

### 3.1 CBF1 expression

**Fig. 1:**
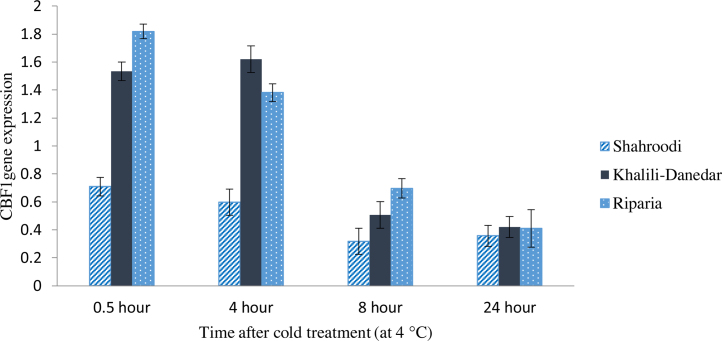
CBF1 expression in three grape cultivars (“Shahroodi”, “Khalili-Danedar” and *V. riparia*) at different times after cold treatment.

Results of ANOVA showed significant variation in CBF1 expression over time after cold treatment for all studied cultivars (Table 2). Expression level of CBF1 increased at early minutes after exposing plants to 4 °C in *V. riparia* and “Khalili-Danedar”, while in “Shahroodi” this increment was not observed till the end of the day. *V. riparia* and “Khalili-Danedar” showed the highest levels of CBF1 expression about 0.5 h and 4 h after cold treatment (Fig. 1). Decreasing trend of CBF1 expression started 8 h after cold treatment and there was no significant differences in the expression level of CBF1 among three studied cultivars, the day after cold treatment. Expression level of CBF1 among studied cultivars was significantly different after 0.5, 4 and 8 h (Table 2). “Shahroodi” and *V. riparia* had the least and the highest expression levels at most sampling times respectively. Expression of CBF1 between “Khalili-Danedar” and *V. riparia* was not significantly different at 8 h, but they both had significant difference with “Shahroodi”. At 24 h after cold treatment, there were no significant differences among them (Fig. 1).

### 3.2 CBF2 expression

**Fig.2:**
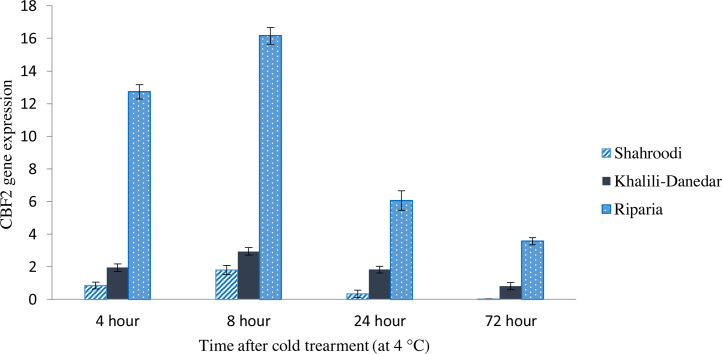
CBF2 expression in three grape cultivars (“Shahroodi”, “Khalili-Danedar” and *V. riparia*) at different times after cold treatment.

Results of ANOVA showed significant variation in CBF2 expression over time after cold treatment for all studied cultivars. Results showed that CBF2 had similar trend but higher expression than CBF1 (Table 2). After cold treatment, the expression level of CBF2 increased drastically in *V. riparia*, while “Khalili-Danedar” and “Shahroodi” showed increment in expression but with lower trend. Three studied cultivars showed the highest expression of CBF2 at 8 h after treatment. Noteworthy, *V. riparia* had expression level of four times than “Khalili-Danedar” and eight times than “Shahroodi”. Also “Shahroodi” showed significantly lower expression than “Khalili-Danedar”, and this gene was not detectable in this cultivar three days after cold treatment. Decreasing trend started eight hours after cold treatment and continued till the third day. To reach the highest expression level, several hours is needed for this gene, but at early minutes the CBF1 showed its highest expression and at the next stage CBF2 started to activated downstream COR genes (Fig. 2). It seems that at the steady state of cold conditions, the first group of transcription factor do not surmount the cell necessity for cold stress acclimation, so the second group of transcription factors start to express.

### 3.3 CBF3 expression

**Fig. 3:**
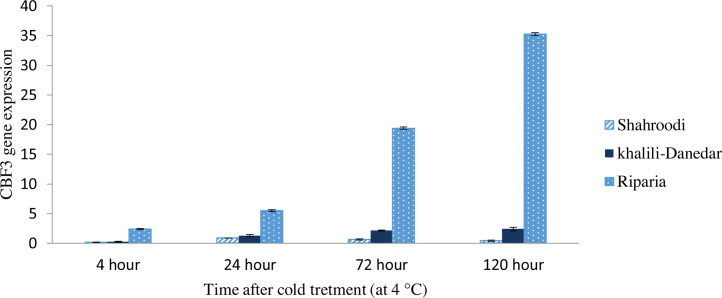
CBF3 expression in three grape cultivars (“Shahroodi”, “Khalili-Danedar” and *V. riparia*) at different times after cold treatment.

CBF3 gene also acts as a transcription factor for induction of downstream genes. ANOVA analysis of CBF3 showed that its expression was significantly different at 1% of probability and increased from the first day at three studied grape cultivars and reached to its highest expression at fifth day after cold treatment (Table 2). Increasing trend of expression continued till the fifth day and afterward presumably decreasing trend happened. In comparison with the CBF1 and CBF2, this gene had higher expression in the *V. riparia*. In the highest expression stage in *V. riparia*, CBF3 showed the expression level of 35 times higher than the reference gene. Altogether, CBF3 expression did not show predominant increase in the Iranian cultivars, and there were no significant differences between these two cultivars till one day after cold treatment. However, CBF3 expression increased in the “Khalili-Danedar” and this cultivar had significantly higher expression than “Shahroodi” from the third day. It is noteworthy that “Khalili-Danedar” had significantly lower expression than *V. riparia*. Quantitative analysis showed that CBF3 expression trigger after CBF2 and steady state of this gene expression is necessary for acclimation to the cold that induced by CBF genes chain (Fig. 3).

In the *V. riparia* species (clearly) and in the “Khalili-Danedar” (with a lower degree) increasing in the expression of CBF3 started a day after cold treatment and did not overlap with CBF2 expression.

### 3.4 CBF4 expression

**Fig. 4:**
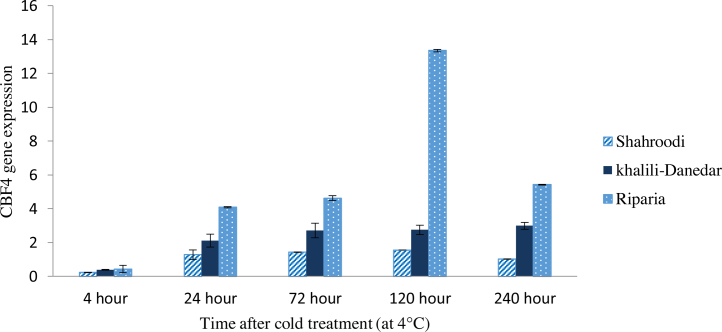
CBF4 expression in three grape cultivars (“Shahroodi”, “Khalili-Danedar” and *V. riparia*) at different times after cold treatment.

Results of ANOVA analysis about CBF4 in the studied cultivars indicated that expression of this gene was significantly different at different sampling times and increased from the first day and continued to the tenth day after cold treatment (Table 3). The highest expression of CBF4 was recorded from *V. riparia* and afterward “Khalili-Danedar”. *V. riparia* showed increasing trend till the fifth day and at the tenth day the expression level decreased. “Khalili-Danedar” also showed higher expression in the response to the cold treatment, but with lower degree compared with *V. riparia*, and the increasing trend about this cultivar remained till the tenth day. There were significant differences in the expression level of CBF4 in *V. riparia* between different time periods of sampling, but in the “Khalili-Danedar” these differences were not visible at all times. Though having some expression till the tenth day, “Shahroodi” did not showed significantly increasing expression in responses to the cold treatment (Fig. 4). “Shahroodi” had significantly lower expression of CBF4 compared to the *V. riparia* and “Khalili-Danedar”.

## 4 Discussion

### 4.1 CBF1 is the first activated gene

In the present study, results of CBF1 expression in “Khalili-Danedar” and *V. riparia* were in agreement with the previous reports. At initial minutes after cold treatment, the first transcription factor (CBF1) that is necessary for activation of downstream genes (COR) will be activated to produce vital proteins for plant acclimation with likely low temperature. According to some reports, CBF1 expression was increased 15 min after cold treatment (Gilmour et al., 1998, Stockinger et al., 1997). Also, Cold treated (5 °C) transgenic Arabidopsis showed increased expression of CBF1 at early hours, and this expression level was higher compared with controlled ones (Mckhann Grey et al., 2008). Results of similar experiment on eucalyptus indicated that expression of two CBF1 alleles (CBF1a and CBF1b) started 0.5 h after cold treatment (4 °C) and reached to the highest amount at 2 h after treatment. Also CBF1a had higher expression than CBF1b (Kayal et al., 2006). Also, in tomato, transcripts of LeCBF1 increased quickly in response to the cold treatment (Jaglo et al., 2001). In similar experiment on tomato, expression of CBF1 in two light periods (constant light and photoperiod of 16 h light and 8 h dark) was investigated. In the constant light, CBF1 transcript was observed 2 h after cold treatment, but after 24 h there were no differences between treated and control plants. In the photoperiod of 16 h light and 8 h dark, the highest expression was observed at 16 h after cold treatment, but decreased after 24 h. However, general expression was higher in the constant light condition compared to the dark and light photoperiod. Another experiment on CBF1 was conducted on a two year-old grapevine “chardonnay” (Xiao et al., 2006). They reported that expression of CBF1 started in a short time after cold treatment, continued 8 h afterwards and then decreased after 12 h (Xiao et al., 2006). So it is reasonable that as time passing, some cold responsive genes are activated. In most cases, CBF1 acts as the first activated gene in plants that trigger a consecutive expression of cold responsive genes. This consecutive expression of genes is the best defensive system against cold stress.

### 4.2 CBF2 activation interference with CBF1 and CBF3 gene

Mckhann et al. (2008) reported that CBF2 expression was significantly higher (four times) than CBF1 and showed higher expression in the resistance Arabidopsis cultivars than susceptible ones. Results of Xiao et al. (2006) indicated that the higher expression of CBF2 was recorded at 2–12 h after cold treatment and the expression of this gene was very low at 2 days after treatment. Results of the present study were in agreement with the previous reports. CBF2 expression in *Vitis amurensis* species (cold resistance) started approximately 2 h after cold treatment (4 °C) and this species had higher expression than *V. vinifera* “Manicure-Finger” (cold susceptible). According to the previous reports on Arabidopsis, expression of AtCBF2 down regulate the expression of AtCBF1 and AtCBF3, as *cbf2* mutant of Arabidopsis showed higher tolerance to the cold stress treatment. In response to the cold stress treatment, AtCBF2 induces after induction of AtCBF1 and AtCBF3 (Novillo et al., 2004). Another experiment on Arabidopsis indicated that AtCBF1 and AtCBF3 have a direct effect on cold resistance, while AtCBF2 act indirectly and activate other transcription factors in response to cold treatment (Novillo et al., 2007). Studies on tomato showed that, unlike Arabidopsis and grape, expression of LeCBF2 was not detectable after cold treatment, and this was attributed to the lack of regulatory regions on this gene's promoter to induce by cold treatment (Gilmour et al., 1998). Transgenic Arabidopsis plant using VvCBF2 showed better tolerance not only to the cold treatment, but also to other stresses such as salinity and drought (Kobayashi et al., 2012). From these observations, it may be deduced that high level of CBF2 expression is require to down regulate CBF1 and CBF3 genes. This gene acts as the connection point of CBF genes, and control the level of CBF1 and CBF3 expression.

### 4.3 CBF3 expression is activated by ICE1

The increasing trend of CBF3 expression was observed when the decreasing trend of CBF2 expression started. This may be due to the down regulatory effect of CBF2 gene on the two other transcription factors. CBF3 expression is affected by ICE1, another transcription factors, whose products regulate the expression of CBF3 by binding to the appropriate sequences of CBF3 promoter (Heather et al., 2006). As indicated previously, similar to CBF1, CBF3 acts directly on cold acclimation genes (Novillo et al., 2007). Studies on susceptible and tolerant Arabidopsis plants revealed that in comparison to the CBF1 and CBF2, the expression of CBF3 was higher in the tolerant cultivars (Mckhann et al., 2008). Results of quantitative expression of this gene in the two studied *vinifera* cultivars and *riparia* species were in agreement with Xiao et al., (2006). These authors have reported that the expression of CBF3 started from the second day and reached to the highest at fifth and seventh days (Xiao et al., 2006).

### 4.4 CBF4 expression continued for longer time than othet CBFs

According to results, “Shahroodi” had lower expression of CBF4 compare to the *V. riparia* and “Khalili-Danedar”. It seems that susceptibility of the “Shahroodi” in the cold conditions could be attributed to the lower ability of this cultivar to activate CBFs and downstream genes and afterward synthesis the cold toleration proteins, therefore this cultivar showed higher susceptibility to the cold than *V. riparia*.

In the mature tissues, VrCBF4 expression continued for several days, that is representative of a particular role of this gene in the stressful acclimation. Biological age did not affect the expression of CBF4, and the transcript of this gene in the young and old leaves of *V. vinifera* as well as *V. riparia* grape species was detected for several days (Xiao et al., 2008). Long last expression of this gene was similar to the BNCBF17 in cabbage (Gao et al., 2002), HvCBF7 in barley (Skinner et al., 2005), and EguCBF1b in eucalyptus (Kayal et al., 2006). There are similar sequences in the promoter of two CBF1 and CBF4 genes, that protein product of ICEr1 gene bind to that and induce this gene not only by cold but also by other stresses (Moody, 2009). CBF4 showed longer time of expression compared with the other studied CBF genes, this could be attributed to the different length or active binding site of its promoter that induce the expression of this gene for more times. In contrast to the Arabidopsis plant that the expression of this gene is limited to the drought and ABA stresses (Haake et al., 2002), in the grape, cold treatment also induces the expression of CBF4. The comparison of the transgenic Arabidopsis plants having grape’s CBF1 and CBF4 genes showed that *Vr*CBF4 transgenic lines were more resistance to the −7 °C cold treatment (Siddiqua and Nassuth, 2011).

## 5 Conclusion

The quantitative analysis of four CBF4 genes showed that two Iranian cultivars and *V. riparia* species had approximately similar trend for each transcription factors. As prospected, the expression of these transcription factors increased after cold treatment and then decreased after a period of time. This trend was expected because this group of transcription factors induces the activation of downstream COR genes. The protein products of downstream genes (COR genes) is recognized as an essential part of plant acclimation to the stressful conditions. Therefore, after some times, these transcription factors activate the target genes adequately, protein products of these genes synthesis according to the cell requirement, and presumably the products of COR genes trigger a feedback pathway to control CBF genes expression. The expression of these transcription factors started minutes after cold treatment (CBF1) and continued for several hours (CBF2) and even till the tenth day (CBF4). The expression of these genes are required for plant acclimation and showed the plant efforts from the early minutes of stress to tolerate the new conditions. Comparison between *V. riparia* and *V. vinifera* species showed that, as expected, *V. riparia*, which is endemic to the cold regions, had higher expression for all transcription factors. Among two studied Iranian cultivars, “Khalili-Danedar” had significantly higher expression than “Shahroodi”. According to these results, it is expected that “Shahroodi” cultivar be more susceptible to the winter freezing compared with the two other studied grape cultivars.

## Figures and Tables

**Table 1:** Sequences of specific primers that were used for qPCR experiment and their amplicon sizes on grapevine cDNA.

Gene	Primer sequences	Amplicon size	Anealing temperature (°C)
CBF1	F:AGAGAAGGTTGGAGATGGTTCA R:CAGGTGGAGTAAGGAGCAAAC	142 bp	60
CBF2	F:CTGCTTCTTCCGACTCTC R:GCACTTCACTCACCCATTTGTT	125 bp	60
CBF3	F:AAGTGCGGGATCCCAAAACC R:GGAGTCGGGGAAATTGAGC	131 bp	60
CBF4	F:ACCCTCACCCGCTCGTATG R:CCGCGTCTCCCGAAACTT	128 bp	60
EF1α	F:CGGGCAAGAGATACCTCAAT R:AGAGCCTCTCCCTCAAAAGG	84 bp	60

**Table 2:** Analysis of variance for CBF1, CBF2 and CBF3 genes in different grape cultivars at various sampling time after cold treatment.

Mean square				
Source	Degree of freedom	CBF1	CBF2	CBF3
Genotype	2	5.997**	21.188**	12.905**
Sampling time	3	0.549**	2.854**	0.836**
Genotype × sampling time	6	0.156**	3.630**	0.290**
Error	24	0.009	0.004	0.019
CV%	–	8.031	4.986	11.698

** Significant at 1% level of probability.

**Table 3:** Analysis of variance for CBF4 gene in different grape genotypes at various sampling time after cold treatment.

Mean square		
Source	Degree of freedom	CBF4
Genotype	2	72.929**
Sampling time	4	2.642**
Genotype × sampling time	8	3.060**
Error	30	0.014
CV%	–	8.374

** Significant at 1% level of probability.

## References

[bib0005] Agarwal P., Agarwal K., Reddy P., Sopory S.K. (2006). Role of DREB transcription factors in abiotic and biotic stress tolerance in plants. Plant Cell Rep..

[bib0010] Century K., Reuber T.L., Ratcliffe O.J. (2008). Regulating the regulators: the future prospects for transcription-factor-based agricultural biotechnology products. Plant Physiol..

[bib0015] Chinnusamy V., Schumaker K., Zhu J.K. (2004). Molecular genetic perspectives on cross-talk and specifcity in abiotic stress signalling in plants. J. Exp. Bot..

[bib0020] Choi D.W., Rodriguez E.M., Close T.J. (2002). Barley Cbf3 gene identification, expression pattern, and map location. Plant Physiol..

[bib0025] Dubouzet J.G., Sakuma Y., Ito Y., Kasuga M., Dubouzet E.G., Miura S. (2003). OsDREB genes in rice, *Oryza sativa* L., encode transcription activators that function in drought-, highsalt- and cold-responsive gene expression. Plant J..

[bib0030] Fennell A. (2004). Freezing tolerance and injury in grapevines. J. Crop Improv..

[bib0035] Gao M.J., Allard G., Byass L., Flanagan A.M., Singh J. (2002). Regulation and characterization of four CBF transcription factors from *Brassica napus*. Plant Mol. Biol. Rep..

[bib0040] Gilmour S.J., Zarka D.G., Stockinger E.J., Salazar M.P., Houghton J.M., Thomashow M.F. (1998). Low temperature regulation of Arabidopsis CBF family of AP2 transcriptional activators as an early step in cold-induced COR gene expression. Plant J..

[bib0045] Haake V., Cook D., Riechmann J.L., Pineda O., Thomashow M.F., Zhang J.Z. (2002). Transcription factor CBF4 is a regulator of drought adaptation in Arabidopsis. Plant Physiol..

[bib0050] Heather A., Buskirk V., Thomashow M. (2006). Arabidopsis transcription factors regulating cold acclimation. Physiol. Plant.

[bib0055] Jaglo K.R., Kleff S., Amundsen K.L., Zhang X., Haake V., Zhang J.Z., Deits T., Thomashow M.F. (2001). Components of the Arabidopsis C-repeat/dehydration-responsive element binding factor cold-response pathway are conserved in *Brassica napus* and other plant species. Plant Physiol..

[bib0060] Jiang F., Wang F., Wu Z., Li Y., Shi G., Hu J., Hou X. (2011). Components of the Arabidopsis CBF cold-response pathway are conserved in non-heading Chinese Cabbage. Plant Mol. Biol. Rep..

[bib0065] Kayal E.W., Navarro M., Marque G., Keller G., Marque C., Teulieres C. (2006). Expression profile of CBF-like transcriptional factor genes from Eucalyptus in response to cold. J. Exp. Bot..

[bib0070] Kobayashi M., Horiuchi H., Fujita K., Takuhara Y., Suzuki S. (2012). Characterization of grape C-repeat-binding factor 2 and B-box-type zing finger protein in transgenic Arabidopsis plants under stress conditions. Plant Mol. Biol. Rep..

[bib0075] Lata C., Prasad M. (2011). Role of DREBs in regulation of abiotic stress responses in plants. J. Exp. Bot..

[bib0080] Liu Q., Kasuga M., Sakuma Y., Abe H., Miura S., Goda H. (1998). Two transcription factors, DREB1 and DREB2, with an EREBP/AP2 DNA binding domain separate two cellular signal transduction pathways in drought- and low-temperature responsive gene expression, respectively, in Arabidopsis. Plant Cell.

[bib0085] Livak K.J., Schmittgen T.D. (2001). Analysis of relative gene expression data using real-time quantitative PCR and the 2^−ΔΔ^C_T_ method. Methods.

[bib0090] Mckhann H.I., Gery C., Berard A., Leveque S., Zuther E., Hincha D., Mita S.D., Brunel D., Teole E. (2008). Natural variation in CBF gene sequence, gene expression and freezing tolerance in the Versailles core collection of *Arabidopsis thaliana*. BMC Plant Biol..

[bib0095] Moody A.M. (2009). Molecular aspects of vitis CBF gene activation. Msc Thesis.

[bib0100] Mullins M.G., Bouquet A., Williams L.E. (1992). Biology of the Grapevine.

[bib0105] Navarro M., Marque G., Ayax C., Keller G., Borges J.P., Marque C., Teulieres C. (2009). Complementary regulation of four eucalyptus CBF genes under various cold conditions. J. Exp. Bot..

[bib0110] Novillo F., Medina J., Salinas J. (2007). Arabidopsis CBF1 and CBF3 have a different function than CBF2 in cold acclimation and define different gene classes in the CBF regulon. Proc. Natl. Acad. Sci. U. S. A..

[bib0115] Novillo F., Alonso J.M., Ecker J.R., Salinas J. (2004). CBF2/DREB1C is a negative regulator of CBF1/DREB1B and CBF3/DREB1A expression and plays a central role in stress tolerance in Arabidopsis. Proc. Natl. Acad. Sci. U. S. A..

[bib0120] Puhakainen T., Li C., Boije-Malm M., Kangasjrvi J., Heino P., Palva E.T. (2004). Short-day potentiation of low temperatureinduced gene expression of a C-repeat-binding factor-controlled gene during cold acclimation in silver birch. Plant Physiol..

[bib0125] Qin Q., Liu J., Zhang Z., Peng R., Xiong A., Yao Q., Chen J. (2007). Isolation, optimization, and functional analysis of the cDNA encoding transcription factor OsDREB1B in *Oryza Sativa* L. Mol. Breed..

[bib0130] Reid K.E., Olsson N., Schlosser J., Peng F., Lund S.T. (2006). An optimized grapevine RNA isolation procedure and statistical determination of reference genes for real-time RT-PCR during berry development. BMC Plant Biol..

[bib0135] Sakuma Y., Liu Q., Dubouzet J.G., Abe H., Shinozaki K., Yamaguchi-Shinozaki K. (2002). DNA-binding specificity of the ERF/AP2 domain of Arabidopsis DREBs, transcription factors involved in dehydration- and cold-inducible gene expression. Biochem. Biophys. Res. Commun..

[bib0140] Shinozaki K., Yamaguchi-Shinozaki K. (2007). Gene networks involved in drought stress response and tolerance. J. Exp. Bot..

[bib0145] Siddiqua M., Nassuth A. (2011). Vitis CBF1 and CBF4 differ in their effect on Arabidopsis abiotic stress tolerance, development and gene expression. Plant Cell Environ..

[bib0150] Skinner J.S., von Zitzewitz J., Szucs P., Marquez-Cedillo L., Filichkin T., Amundsen K., Stockinger E.J., Thomashow M.F., Chen T.N.N., Hayes P.M. (2005). Structural, functional, and phylogenetic characterization of a large CBF gene family in barley. Plant Mol. Biol. Rep..

[bib0155] Stockinger E.J., Gilmour S.J., Thomashow M.F. (1997). Arabidopsis thaliana CBF1 encodes an AP2 domain-containing transcription activator that binds to the C-repeat/DRE, a cis-acting DNA regulatory element that stimulates transcription in response to low temperature and water deficit. Proc. Natl. Acad. Sci. U. S. A..

[bib0160] Thomashow M.F. (2001). So what’s new in the field of plant cold acclimation?. Plant Physiol..

[bib0165] Wang Q.J., Xu K.Y., Tong Z.G., Wang S.H., Gao Z.H., Zhang J.Y. (2010). Characterization of a new dehydration responsive element binding factor in central arctic cowberry. Plant Cell Tissue Organ.

[bib0170] Wang Q., Guan Y., Wu Y., Chen H., Chen F., Chu C. (2008). Overexpression of a rice OsDREB1F gene increases salt, drought, and low temperature tolerance in both Arabidopsis and rice. Plant Mol. Biol. Rep..

[bib0175] Welling A., Palva T. (2008). Involvement of CBF transcription factors in winter hardiness in Birch. Plant Physiol..

[bib0180] Xiao H., Tattersall E.A.R., Siddiqua M.K., Cramer G., Nassuth A. (2008). CBF4 is a unique member of the CBF transcription factor family of *Vitis vinifera* and *Vitis riparia*. Plant Cell Environ..

[bib0185] Xiao H., Siddiqua M., Braybrook S., Nassuth A. (2006). Three grape CBF/DREB1 genes respond to low temperature, drought and abscisic acid. Plant Cell Environ..

[bib0190] Yang W., Liu X.D., Chi X.J., Wu C.A., Li Y.Z., Song L.L. (2011). Dwarf apple MbDREB1 enhances plant tolerance to low temperature, drought, and salt stress via both ABA-dependent and ABA-independent pathways. Planta.

[bib0195] Zhang X., Fowler S.G., Cheng H., Lou Y., Rhee S.Y., Stockinger E.J., Thomashow M.F. (2004). Freezing-sensitive tomato has a functional CBF cold response pathway, but a CBF regulon that differs from that of freezing-tolerant Arabidopsis. Plant J..

